# Diethylnitrosamine Increases Proliferation in Early Stages of Hepatic Carcinogenesis in Insulin-Treated Type 1 Diabetic Mice

**DOI:** 10.1155/2018/9472939

**Published:** 2018-04-23

**Authors:** A. S. Arboatti, F. Lambertucci, M. G. Sedlmeier, G. Pisani, J. Monti, M. de L. Álvarez, D. E. A. Francés, M. T. Ronco, C. E. Carnovale

**Affiliations:** ^1^Instituto de Fisiología Experimental (IFISE-CONICET), Cátedra de Fisiología, Facultad de Ciencias Bioquímicas y Farmacéuticas, UNR, Suipacha 570, 2000 Rosario, Argentina; ^2^Cátedra de Morfología, Facultad de Ciencias Bioquímicas y Farmacéuticas, UNR, Suipacha 570, 2000 Rosario, Argentina

## Abstract

Diethylnitrosamine (DEN) induces hepatocarcinogenesis, increasing mitotic hepatocytes and leading to chronic inflammation. In addition, type 1 diabetes mellitus (T1DM) is also characterized by a proinflammatory state and by requiring insulin exogenous treatment. Given the association of diabetes, insulin treatment, and cell proliferation, our specific goal was to determine whether the liver in the diabetic state presents a greater response to DEN-induced cell cycle alteration, which is essential for the malignant transformation. Male C57BL/6 mice (four-week-old) were divided into 4 groups: C, C + DEN, T1DM, and T1DM + DEN. Mice were euthanized ten weeks after DEN injection. DEN per se produced an increase in liver lipid peroxidation levels. Besides, in T1DM + DEN, we found a greater increase in the proliferation index, in comparison with C + DEN. These results are in agreement with the increased expression observed in cell cycle progression markers: cyclin D1 and E1. In addition, a proapoptotic factor, such as activated caspase-3, evidenced a decrease in T1DM + DEN, while the Vascular Endothelial Growth Factor (VEGF) and the protooncogene p53 showed a higher increase with respect to C + DEN. Overall, the results allow us to highlight a major DEN response in T1DM, which may explain in part the greater predisposition to the development of hepatocarcinoma (HCC) during the diabetic state.

## 1. Introduction

Liver cancer is one of the most common malignancies worldwide. Hepatocellular carcinoma (HCC) accounts for about 80%–90% of all liver cancers and is the fifth most common cause of cancer mortality [1, 2]. Major risk factors for liver cancer include viral hepatitis infection, food additives, alcohol abuse, toxic exposure (aflatoxin-B1 (AFB1) intake from contaminated food), environmental and industrial toxic chemicals, and air and water pollutants [3, 4]. Besides, obesity, metabolic diseases (starting from nonalcoholic fatty liver disease (NAFLD) and nonalcoholic steatohepatitis (NASH)), diabetes, and autoimmune reactions also contribute to HCC development [5–8].

Diethylnitrosamine (DEN) is a well-known potent hepatocarcinogen agent present in tobacco smoke, water, cured and fried meals, cheddar cheese, agricultural chemicals, cosmetics, and pharmaceutical products [9, 10]. Cytotoxicity induced by DEN is critical for malignant transformation [11, 12].

DEN is known to induce damage in many enzymes involved in DNA repair and is normally used to induce liver cancer in experimental animal models [13–16]. Exposure to DEN has also been associated with hepatocellular accumulation of reactive oxygen species (ROS) [17], which may result in oxidative damage to DNA and other nucleophiles, a mechanism that may further enhance DEN-induced hepatocarcinogenesis. Although the coordination of cell replication and apoptosis is maintained in normal liver tissue, the disruption in this balance that takes place in precancerous and cancerous tissues is considered one of the hallmarks of cancer [17]. In this connection, many lines of evidence have demonstrated a close relationship between carcinogenesis and cell cycle regulation. Park et al. (2009) found that cell cycle regulatory proteins were critically involved in hepatic cancer initiation and promotion by DEN. In this regard, DEN induces irreversible hepatocellular carcinogenesis through overexpression of G1/S phase regulatory proteins in rat hepatocytes [18].

Type 1 diabetes mellitus (T1DM) is a serious and growing health problem worldwide and is associated with severe acute and chronic complications that negatively influence both the quality of life and survival of affected individuals. Clinically, altered liver size is seen in both juvenile and adult diabetic patients, which can be the result of alteration in cell number, cell growth, and/or cell death (apoptosis) [19, 20]. The chronic hyperglycemia can directly promote a proinflammatory status, where the increase in cytokines levels can lead to destruction of the pancreatic beta cells and malfunction of the endocrine pancreas [21]. T1DM, which is considered as an autoimmune disease, is influenced by genetic and environmental factors and is characterized by T cells-mediated pancreatic beta cells destruction, which leads to partial or total absence of insulin secretion [22]. T1DM can be replicated in animal models through genetic modifications [23]. Another way to obtain experimental animals with type 1 diabetes involves the administration of diabetogenic chemicals such as Streptozotocin (STZ). It is known that STZ is excreted within 48 h from injection and therefore cannot be a direct effect of oxidative stress [24]. Seven days after STZ injection is the time when acute STZ toxicity in both rats and mice has already disappeared [25–28]. Dombrowski et al. (1997) and Kazumi et al. (1978) confirmed that hepatocarcinogenesis in their STZ diabetic rat model is independent of STZ [29, 30]. Besides, Ichikawa et al. (2010) demonstrated that STZ did not accelerate per se the initiation by DEN in rats [31]. Moreover, Schmezer et al. (1994) reported that STZ did not induce malignant tumors in mouse liver [32]. Regarding insulin treatment, if STZ-induced diabetic mice do not receive exogenous insulin, they can suffer severe and even fatal ketoacidosis [33].

We demonstrated that hyperglycemia increases ROS levels, specifically hydroxyl radicals, which initiate mitochondrial apoptosis either directly and/or by acting through Bcl2 family proteins, thus inducing the formation of the mitochondrial permeability transition pore (MPTP), the release of cytochrome c, and the activation of caspase-3 [34]. In this sense, insulin leads to a decrease of radical hydroxyl levels, which produces a slight reduction of Bax : Bcl-xl ratio, leading to a mild diminution of cytochrome c release from mitochondria to cytosol and a significant decrease in both caspase-3 activity and apoptotic index [35]. Keeping in mind the well-established ability of DEN to induce hepatocarcinogenesis, the T1DM-derived proinflammatory state, and the requirement of continuous insulin exogenous treatment, our specific goal was to determine whether the liver in the diabetic state is more sensitive to DEN-induced cell cycle alteration which was essential for the malignant transformation.

## 2. Materials and Methods

### 2.1. Animals and Experimental Design

Four-week-old male C57BL/6 mice were provided by the School of Medicine, National University of Rosario, and were maintained at the animal facilities of the School of Biochemistry of the National University of Rosario. Animals received humane care according to criteria outlined in the* Guide for the Care and Use of Laboratory Animals* (National Research Council, Washington DC: National Academy Press, 1996). All the experimental protocols were performed according to the Regulation for the Care and Use of Laboratory Animals (Expedient 6109/012 E.C. Resolution 267/02) and approved by the Institutional Animal Use Committee of the National University of Rosario, Argentina.

**Figure 1:**
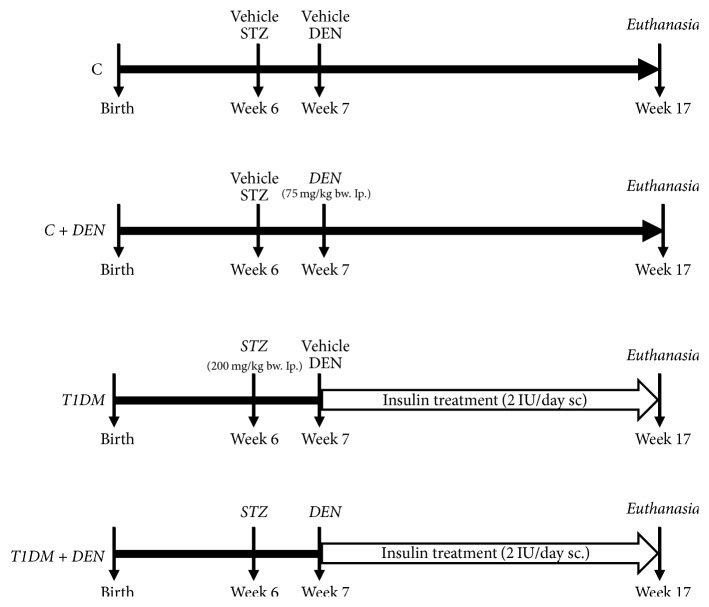
*Experimental protocol. *Four-week-old male C57BL/6 mice were randomly divided into 4 experimental groups (*n* = 5 per group). Mice of group 1 (control group, C) received a single intraperitoneal (i.p.) injection of both buffer citrate at week 6 and saline solution at week 7, vehicles of Streptozotocin (STZ) and DEN, respectively. Animals of group 2 (C + DEN group) were injected with a single dose of DEN (75 mg/kg, i.p.) at week 7 and buffer citrate vehicle of STZ at week 6. Mice of group 3 (diabetic-induced group with insulin treatment, T1DM group) were injected with a single dose of STZ (200 mg/kg bw, i.p.) at week 6 and with saline solution DEN-vehicle at week 7 (one week after STZ injection), and after one week of STZ injection, they were daily injected with insulin (2 UI/day, subcutaneously) divided into two doses: 0.5 UI at 7 a.m. and 1.5 UI at 7 p.m., during ten weeks. Mice of group 4 (T1DM + DEN group) were treated with STZ at week 6 and after one week with a single dose of DEN and with insulin daily (2 UI/day, subcutaneously) divided into two doses: 0.5 UI at 7 a.m. and 1.5 UI at 7 p.m., during ten weeks. Mice were euthanized 10 weeks after saline solution DEN-vehicle or DEN injection.

Mice were randomly divided into 4 experimental groups (*n* = 5 per group), housed in plastic cages, and kept on hardwood bedding in animal facilities with a 12-hour light/dark cycle and controlled temperature (23 ± 2°C) and ventilation. Water and a standard diet were provided ad libitum throughout the experiment. Mice of group 1 (control group, C) received a single intraperitoneal (i.p.) injection of both citrate buffer at week 6 and saline solution at week 7, vehicles of STZ (diabetogenic agent) and DEN (carcinogenic agent), respectively. Animals of group 2 (C + DEN group) were injected with a single dose of DEN (75 mg/kg, i.p.) [36] at week 7 and citrate buffer vehicle of STZ at week 6 (one week before DEN injection). Mice of group 3 (diabetic-induced group with insulin treatment, T1DM group) were injected with a single dose of STZ (200 mg/kg bw, i.p.) [37] at week 6 and with saline solution DEN-vehicle at week 7 (one week after STZ injection), and after one week of STZ injection, they were daily injected with insulin (2 UI/day subcutaneously); the dose was adjusted with the measurement of blood glucose levels, divided into two doses: 0.5 UI at 7 a.m. and 1.5 UI at 7 p.m., during ten weeks. Mice of group 4 (T1DM + DEN group) were treated with STZ at week 6 and after one week with a single dose of DEN and with insulin daily (2 UI/day, subcutaneously) divided into two doses: 0.5 UI at 7 a.m. and 1.5 UI at 7 p.m., during ten weeks. The diabetic status was demonstrated by measuring of the blood sugar levels one week after STZ injection (476.83 ± 10.53 mg/dl); at this time, it was demonstrated that the hepatic toxic effects disappear [26, 27]. Mice were euthanized ten weeks after saline solution DEN-vehicle or DEN injection with a mixture of ketamine (100 mg/kg bw) and xylazine (10 mg/kg bw). Livers were promptly removed, frozen in liquid nitrogen, and stored at −70°C until analytical assays were performed. Samples of liver tissue were fixed, processed, and embedded in paraffin for immunohistochemistry assays. A scheme of the experimental protocol is shown in Figure 1.

### 2.2. Lipid Peroxidation Assay

Lipid peroxidation (LPO) levels were determined as indirect measurements of ROS [35]. A fraction of liver tissue was homogenized in 1 mL of saline solution and centrifuged at 2000 ×g at 4°C for 5 min. Thiobarbituric acid reactive substances (TBARS) assays measure the total level of oxidized lipids based on the reaction of malondialdehyde (MDA), one of the end products of lipid peroxidation, with thiobarbituric acid (TBA) [38]. An aliquot obtained as previously described was mixed with 20% acetic acid, 0.70% TBA, and 8.1% SDS. This system was heated at 95°C for 1 hour and the amount of aldehydic products generated by lipid peroxidation (LPO) was determined by HPLC [39], and the amount of TBARS was expressed as percentage of C group.

### 2.3. Determination of Proliferative Index (PI)

To investigate modifications in proliferation activity among the experimental groups, liver slides were examined by immunohistochemical staining with anti-proliferating cell nuclear antigen (PCNA) antibody, determined by the method of Greenwell et al. [40]. The PCNA proliferative index is defined as the number of proliferative cells (in G1, S, G2, and M phases) per 100 hepatocytes counted in ten high-power fields [41].

PCNA, as a naturally occurring cell marker of proliferating cells, offers an alternative method for investigating cell proliferation. Molecular studies indicate that the synthesis of PCNA is initiated in the nucleus in late G1 phase and continues during the S phase. The different staining patterns recognized in this study are believed to reflect individual phases of the cell cycle. Cells expressing no staining in the nucleus or cytoplasm are expected to be quiescent Go phase cells. Minimal nuclear staining would be consistent with G1 phase cells. Deep red nuclear staining is characteristic of S phase. G2 cells present speckled nuclear and cytoplasmic staining. In mitosis (M) the nucleoplasm and cytoplasm coalesce with the loss of nuclear boundaries. This could account for the diffuse speckled cytoplasmic staining specifically observed in all actively mitotic cells [42, 43].

### 2.4. Immunoblot Assays

Tissue samples were homogenized in 300 mM sucrose with protease inhibitors. Cytosolic, mitochondrial, and nuclear extracts were prepared as described previously [44]. Protein concentration was determined by the Lowry method [45]. Equal amounts of protein were resolved by 12% sodium dodecyl sulfate-polyacrylamide gel electrophoresis (SDS-PAGE) and electroblotted onto polyvinyl difluoride (PVDF) membranes (PerkinElmer Life Sciences, Inc., Boston, MA). Membranes were blocked with PBS-Tween-10% nonfat milk, washed, and incubated overnight at 4°C with primary antibodies (cyclin D1 (H-295): sc-753, cyclin E1 (E-4): sc-25303, Bcl-xL (H-5) sc-8392, VEGF (147): sc-507, cytochrome c (A-8): sc-13156, p53 (DO-1): sc-126, PCNA (PC10): sc-56 Santa Cruz Biotechnology, Bax #2772, Cell Signaling Technology). Finally, membranes were incubated with peroxidase conjugated secondary antibodies and bands were detected by enhanced chemiluminescence (ECL) detection system (Thermo Fisher Scientific, Rockford, IL). The immunoreactive bands were quantified by densitometry using the ImageJ software (https://imagej.nih.gov/). Equal loading and protein transference were checked by detection in mitochondrial fraction by prohibitin, cytosolic fraction by GAPDH, and nuclear fraction by* Ponceau S* staining of the membranes. According to the bibliography, nuclear loading controls are modified in association with diabetes or HCC, and thus* Ponceau S* staining is the most reliable loading control in nucleus in STZ or DEN-treated mice livers [46, 47].

### 2.5. Statistical Analysis

Data are expressed as means ± SEM. Significance was determined by Student's* t*-test; one-way or two-way analysis of variance followed by Tukey's test was applied wherever necessary. Differences were considered to be statistically significant when *p* < 0.05.

## 3. Results

### 3.1. Analysis of Lipid Peroxidation

**Figure 2:**
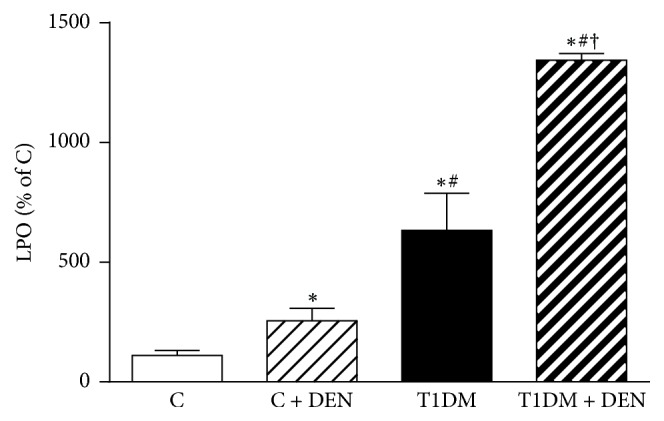
*Lipid peroxidation (LPO). *LPO was determined by quantification of the amount of thiobarbituric acid reactive substances (TBARS) by HPLC. Data are expressed as percentage of C group and mean ± SEM; *n* = 5. ^*∗*^*p* < 0.05 versus C; ^#^*p* < 0.05 versus C + DEN group; and ^†^*p* < 0.05 versus T1DM.

DEN treatment induced an increase of 102% in the generation of TBARS in C + DEN as compared to control animals, highlighting the known oxidizing effect of DEN [48] (Figure 2). Besides, an increase of 112% in TBARS was observed in T1DM + DEN group as compared to T1DM group. The T1DM + DEN group showed significant differences when compared to C + DEN, evidencing that T1DM has an increased LPO level per se, and the treatment with DEN further increases LPO.

### 3.2. Effect of DEN on the Proliferative Status

**Figure 3:**
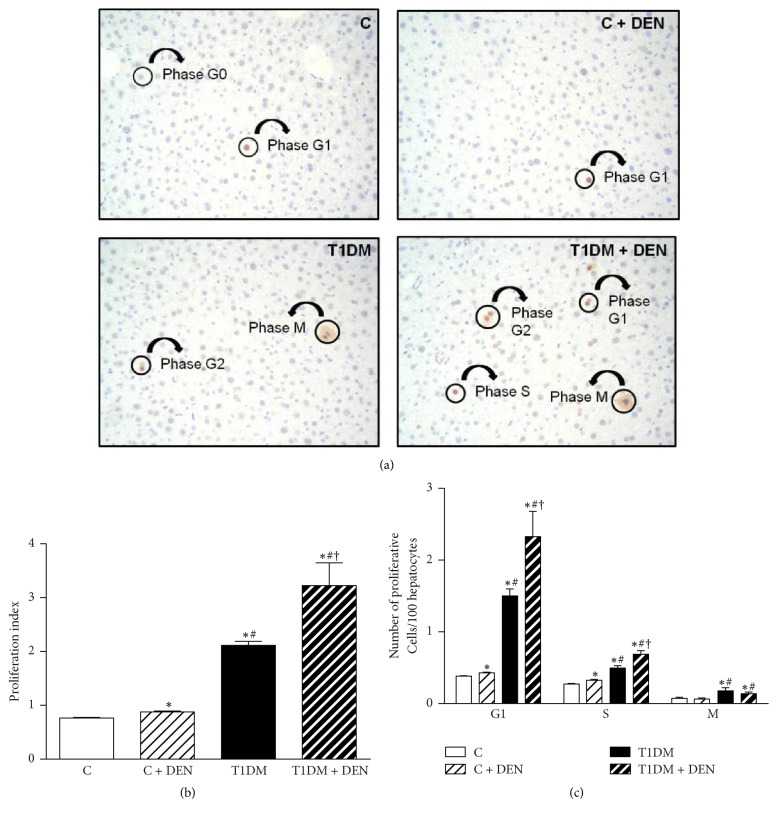
*Proliferative status of liver*. (a) Representative images of proliferating cell nuclear antigen- (PCNA-) positive cells obtained by optical microscopy (objective: 20x; ocular: 10x). (b) Proliferative index (number of proliferative cells in each phase/100 hepatocytes) levels. (c) Determination of the percentage of hepatocytes in each phase of the cell cycle. Data are expressed as mean ± SEM; *n* = 5. ^*∗*^*p* < 0.05 versus C; ^#^*p* < 0.05 versus C + DEN group; and ^†^*p* < 0.05 versus T1DM.

Figure 3(a) shows representative images for immunohistochemical detection of PCNA-positive cells. As shown in Figure 3(b), livers of C + DEN group exhibited an increase of cell replication in comparison with C group, which show the characteristic quiescent status of normal adult liver [49]. When we analyzed the differences between the T1DM mice treated with or without DEN, we observed an increase of 52% in the PI associated with DEN treatment. This change is 3.5-fold higher than that observed between nondiabetic untreated and DEN-treated mice, thus suggesting a major proliferative profile in sensitized livers due to diabetic status. Additionally, we determined the percentages of hepatocytes in each phase of the cell cycle (Figure 3(c)). Significantly higher percentages of cells in G1 were observed in T1DM + DEN group when compared to both C + DEN (+454%) and T1DM (+55%). In line with this, a similar pattern of increase in the number of cells in S phase was found in T1DM + DEN (+115% and +39%, resp.), suggesting higher entrance of cells into the cell cycle. Accordingly, T1DM + DEN group showed an increase in the percentage of cells in M phase with respect to C + DEN (+139%).

### 3.3. Effect of DEN on the Expression of Cell Cycle Regulatory Proteins

**Figure 4:**
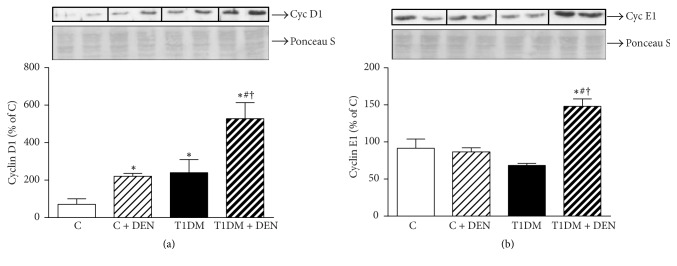
*Expression of cell cycle regulatory proteins.* Markers of cell cycle progression were determined by Western blot in nuclear fraction: (a) cyclin D1 and (b) cyclin E1 expression.* Ponceau S* was probed as loading control in nuclear fraction. Data are expressed as percentage of C group and mean ± SEM; *n* = 5. ^*∗*^*p* < 0.05 versus C; ^#^*p* < 0.05 versus C + DEN group; and ^†^*p* < 0.05 versus T1DM.

As cyclin E1 and cyclin D1 have an important role in the progression of cell cycle from phase G1 to phase S, we evaluated both proteins' nuclear levels by Western blot. Analyses revealed an increase of cyclin E1 and cyclin D1 nuclear localization in T1DM + DEN group when compared to T1DM group (+120% and +116%, resp.) (Figures 4(a) and 4(b)). Also, T1DM + DEN group showed a notable increase in the levels of these proteins with respect to C + DEN group (+139% and +73%, resp.).

### 3.4. Effect of DEN on Programmed Cell Death Pathways

**Figure 5:**
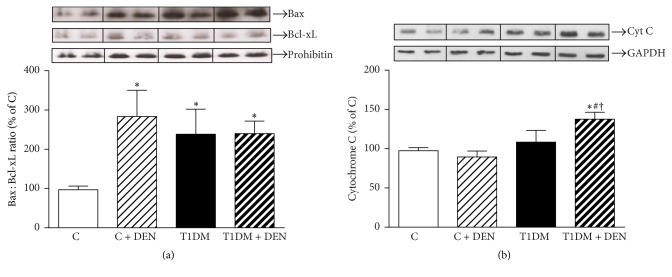
*Apoptotic cell death*. (a) Mitochondrial levels of proapoptotic Bax and antiapoptotic Bcl-xL proteins were analyzed by Western blot. After densitometric quantitation, Bax : Bcl-xL ratio was calculated, and results were expressed as percentage of C group. Prohibitin was probed as loading control in mitochondrial fraction. (b) Release of cytochrome c was determined by Western blot in cytosolic extracts from each experimental group. GAPDH was probed as loading control in cytosolic fraction. Data are expressed as percentage of C group and mean ± SEM; *n* = 5. ^*∗*^*p* < 0.05 versus C; ^#^*p* < 0.05 versus C + DEN group; and ^†^*p* < 0.05 versus T1DM.

Bax : Bcl-xL could reflect accurately the status of apoptosis, since the fate of the cells (survival or death) is largely dependent on the mitochondrial Bax : Bcl-xL ratio [50, 51]. We measured mitochondrial Bax and Bcl-xL levels.  Figure 5(a) shows that mitochondrial Bax : Bcl-xL ratio was significantly increased in C + DEN, T1DM, and T1DM + DEN relative to C, indicating that, in both DEN treatment and diabetic state, the liver is promoted to a proapoptotic state. Besides, cytochrome c cytosolic release shows significant differences in T1DM + DEN with respect to C + DEN and T1DM groups demonstrating slight activation of the apoptotic pathway (Figure 5(b)).

### 3.5. Analysis of Expression Levels of VEGF and Protooncogene p53

**Figure 6:**
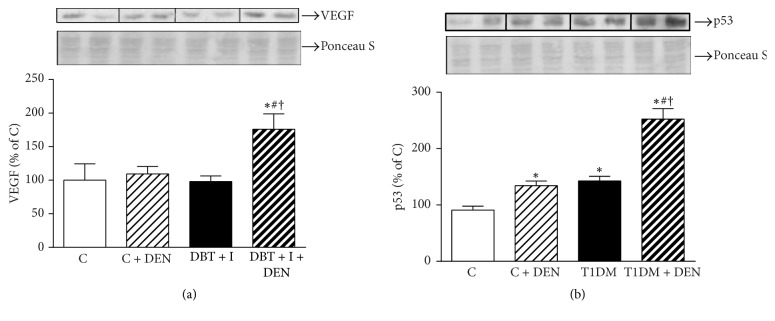
*Expression levels of VEGF and protooncogene p53.* (a) VEGF and (b) p53 proteins were evaluated by Western blot in nuclear extracts.* Ponceau S* was probed as loading control in nuclear fraction. Data are expressed as percentage of C group and mean ± SEM; *n* = 5. ^*∗*^*p* < 0.05 versus C; ^#^*p* < 0.05 versus C + DEN group; and ^†^*p* < 0.05 versus T1DM.

In most hepatocarcinogenesis models, a very long latent period occurs, following initial exposure to the carcinogen. During this latent period, the liver cells are not quiescent. Preneoplastic hepatocytes differ from normal hepatocytes according to the expression of many mediators, some of which are overexpressed, while the expressions of others are decreased [17, 52]. In this regard, we evaluated the effect of DEN on Vascular Endothelial Growth Factor (VEGF) and the protooncogene p53 expression. We found that DEN was able to increase by +64% and +77%, respectively, the expression of these markers in T1DM. Besides, VEGF and p53 proteins were notably increased in T1DM + DEN group with respect to C + DEN group +63% and +88%, respectively (Figures 6(a) and 6(b)). These results evidence a major sensibility to DEN in T1DM group.

## 4. Discussion

The pathogenic equation for T1DM presents a complex interrelation of metabolic, genetic, and environmental factors, as well as inflammatory mediators. Among the latter, it is mostly unclear whether they reflect the disease process or are simply signs of systemic or local responses to the disease [21]. The high incidence of HCC is related to high exposure to known risk factors such as alcohol consumption, cigarette smoking, viral infections, and food contaminants. Many of these factors involve exposure to compounds such as N-nitrosamines that have carcinogenic, mutagenic, and teratogenic properties. DEN is a genotoxic, carcinogenic nitrosamine having its place among N-nitrosodialkylamines [53]. Exposure to DEN has also been associated with hepatocellular accumulation of ROS [48], which may result in oxidative damage to DNA and other nucleophiles, a mechanism that may further enhance DEN-induced hepatocarcinogenesis [54].

Increased generation of ROS and decreased antioxidant enzymes in liver tissues have been reported in many models of DEN-induced hepatocellular carcinoma [55, 56]. Data from the present study revealed that DEN significantly increased TBARS in mice from both groups, control and T1DM groups, thus playing an important role in DEN-induced hepatic damage. It has been reported that ROS play a major role in tumor promotion through interaction with critical macromolecules including lipids, DNA, DNA repair systems, and other enzymes [57]. In this regard, the relationship of factors involved in cell cycle regulation to cancer has been extensively reported [58, 59]. Specifically, Masaki et al. reported the roles of cell cycle-related proteins in spontaneous HCC in Long-Evans Cinnamon rats and suggested that cyclin D1 and cyclin E1 are involved in the transition from normal liver to HCC. The cyclin D1 protooncogene is an important regulator of G1 to S phase transition in numerous cell types from diverse tissues. Moreover, cyclin D1 has also been shown to act as a cofactor for several transcription factors [49]. Studies indicated that cyclin D1 is localized predominantly in the nucleus of asynchronously growing cells [60]. During cell cycle progression, protein levels of cyclin D1 begin to rise early in G1, prior to its rapid nuclear export and degradation within the cytoplasm. Interestingly, the nuclear export and/or degradation of cyclin D1 is required for S phase progression as failure to remove the cyclin results in G1 arrest [61]. Therefore, this protein is considered the “rate-limiting” step in hepatocyte proliferation, suggesting that administration of DEN leads to upregulation of the cell cycle and expression level of cyclin D1 and inhibition of apoptosis and may lead to HCC [18]. In this study, we observed that T1DM group represented a risk factor that increased the sensibility to DEN-induced hepatic cell cycle alteration. In this connection, our results demonstrate that in livers of T1DM mice there is an increase in proliferative rate in accordance with higher levels of proteins involved in cell cycle regulation as cyclin D1 and cyclin E1 which are detected in chronical liver diseases [62]. Our results show that the liver of the T1DM mice is more sensible to the effect of the DEN treatment, thus suggesting that it would favor the cell cycle alteration, which is essential, in the early stage, for the malignant transformation.

Programmed cell death plays an important role in the genesis of cancer. In this regard, several proteins that are structurally related to Bcl-2, an inhibitor of apoptosis, have been identified [63]. Diverse strategies and agents that target specific molecular pathways, as that of triggering a process of cell death, are being evaluated in the treatment of several neoplasms. The homologous Bcl-2 and Bcl-xL proteins can extend cell survival by suppressing apoptosis, whereas the proapoptotic proteins (e.g., Bax, Bak, and Bik) act as dominant cell death inducers when they are overexpressed [51, 64]. We analyzed the expression of Bax and Bcl-xL protein to estimate the apoptosis process [65]. Ours results show that Bax : Bcl-xL ratio did not increase in T1DM + DEN group as compared to T1DM group, and neither versus C + DEN group, but the three groups increase versus control group. Besides, cytochrome c cytosolic release showed a significant difference in T1DM + DEN group as compared to T1DM group and versus C + DEN group. These results allow us to hypothesize that, 10 weeks after administration of DEN (75 mg/kg bw), the hepatocellular response leads to a counterbalance of the apoptotic mechanism with an increase in cell proliferation (more evident in T1DM + DEN group). On the other hand, p53 activation following DNA damage induced by DEN was described as a likely mechanism to induce protumorigenic inflammation in the liver [66]. Although p53 is generally considered as a tumor suppressor, its continuous activation may promote protumorigenic inflammation, especially in livers exposed to agents that cause long-term mutagenic DNA damage such as DEN [66]. In agreement with this, our results show a significant increase of p53 in the DEN-treated groups that were higher in livers of T1DM mice.

VEGF is the main growth factor implicated in angiogenesis. It has been demonstrated by immunohistochemistry that VEGF expression is increased in 36% to 83% of HCC cells [67]. Park et al. [68] demonstrated that VEGF is overexpressed in hepatic foci and tumors developed during DEN-derived carcinogenesis in mice with iron overload. Moreover, Bozova and Elpek showed that intraperitoneal administration of DEN to male Wistar rats induced an increase of VEGF expression [69]. Here, we demonstrate that in the liver DEN is able to induce an increase of VEGF expression, being, again, its expression higher in T1DM group.

Taken together, our results show a greater liver sensitivity to DEN treatment in T1DM group, suggesting that the degree of chronic inflammation, increased ROS levels, and alterations in cell cycle balance derived from insulin treatment, among other factors that are hepatic hallmarks of these mice, could play a fundamental role in its sensitization.

In conclusion, data from this study suggest for the first time that, in T1DM + DEN group, the liver cells are not quiescent and exhibit alterations in the levels of certain proteins involved in cell cycle progression. During a diabetic pathology accompanied by insulin treatment, the exposure to a carcinogen such as DEN could lead to an altered early stage in the hepatocarcinogenesis progression. In this regard, this precondition could represent a risk factor to an accelerated DEN-induced process. In this sense, it becomes highly relevant to understand the pathways involved in the development of the initial phases of HCC in T1DM and therefore find the main targets responsible for the alteration in the cell cycle progression.
